# Global Scale Dissemination of ST93: A Divergent *Staphylococcus aureus* Epidemic Lineage That Has Recently Emerged From Remote Northern Australia

**DOI:** 10.3389/fmicb.2018.01453

**Published:** 2018-07-09

**Authors:** Sebastiaan J. van Hal, Eike J. Steinig, Patiyan Andersson, Matthew T. G. Holden, Simon R. Harris, Graeme R. Nimmo, Deborah A. Williamson, Helen Heffernan, S. R. Ritchie, Angela M. Kearns, Matthew J. Ellington, Elizabeth Dickson, Herminia de Lencastre, Geoffrey W. Coombs, Stephen D. Bentley, Julian Parkhill, Deborah C. Holt, Phillip M. Giffard, Steven Y. C. Tong

**Affiliations:** ^1^Department of Microbiology and Infectious Diseases, Royal Prince Alfred Hospital, Sydney, NSW, Australia; ^2^Global and Tropical Health Division, Menzies School of Health Research, Darwin, NT, Australia; ^3^Australian Institute of Tropical Health and Medicine, Townsville, QLD, Australia; ^4^School of Medicine, University of St. Andrews, Fife, United Kingdom; ^5^Pathogen Genomics, Wellcome Trust Sanger Institute, Cambridge, United Kingdom; ^6^Pathology Queensland Central Laboratory and Griffith University School of Medicine, Queensland Health, Brisbane, QLD, Australia; ^7^Microbiological Diagnostic Unit Public Health Laboratory, Department of Microbiology and Immunology, The University of Melbourne at The Peter Doherty Institute for Infection and Immunity, Melbourne, VIC, Australia; ^8^Institute of Environmental Science and Research, Porirua, New Zealand; ^9^School of Medical Sciences, University of Auckland, Auckland, New Zealand; ^10^Antimicrobial Resistance and Healthcare Associated Infections Reference Unit, National Infection Service, Public Health England, London, United Kingdom; ^11^National Infection Service, Public Health England, Addenbrooke’s Hospital, Cambridge, United Kingdom; ^12^Scottish MRSA Reference Service, Scottish Microbiology Reference Laboratories, Glasgow Royal Infirmary, Glasgow, United Kingdom; ^13^Laboratory of Molecular Genetics, Instituto de Tecnologia Química e Biológica António Xavier, Universidade Nova de Lisboa, Oeiras, Portugal; ^14^Laboratory of Microbiology and Infectious Diseases, The Rockefeller University, New York, NY, United States; ^15^School of Veterinary and Life Sciences, Murdoch University, Murdoch, WA, Australia; ^16^Department of Microbiology, Fiona Stanley Hospital, Perth, WA, Australia; ^17^Victorian Infectious Disease Service, The Royal Melbourne Hospital, and The University of Melbourne at the Peter Doherty Institute for Infection and Immunity, Melbourne, VIC, Australia

**Keywords:** *Staphylococcus aureus*, MRSA, community-associated, ST93, evolution, Aboriginal, Indigenous

## Abstract

**Background:** In Australia, community-associated methicillin-resistant *Staphylococcus aureus* (MRSA) lineage sequence type (ST) 93 has rapidly risen to dominance since being described in the early 1990s. We examined 459 ST93 genome sequences from Australia, New Zealand, Samoa, and Europe to investigate the evolutionary history of ST93, its emergence in Australia and subsequent spread overseas.

**Results:** Comparisons with other *S. aureus* genomes indicate that ST93 is an early diverging and recombinant lineage, comprising of segments from the ST59/ST121 lineage and from a divergent but currently unsampled Staphylococcal population. However, within extant ST93 strains limited genetic diversity was apparent with the most recent common ancestor dated to 1977 (95% highest posterior density 1973–1981). An epidemic ST93 population arose from a methicillin-susceptible progenitor in remote Northern Australia, which has a proportionally large Indigenous population, with documented overcrowded housing and a high burden of skin infection. Methicillin-resistance was acquired three times in these regions, with a clade harboring a staphylococcal cassette chromosome *mec* (SCC*mec*) IVa expanding and spreading to Australia’s east coast by 2000. We observed sporadic and non-sustained introductions of ST93-MRSA-IVa to the United Kingdom. In contrast, in New Zealand, ST93-MRSA-IVa was sustainably transmitted with clonal expansion within the Pacific Islander population, who experience similar disadvantages as Australian Indigenous populations.

**Conclusion:** ST93 has a highly recombinant genome including portions derived from an early diverging *S. aureus* population. Our findings highlight the need to understand host population factors in the emergence and spread of antimicrobial resistant community pathogens.

## Introduction

Over the past two decades multiple clones of community-associated methicillin-resistant *Staphylococcus aureus* (CA-MRSA) have emerged globally (DeLeo et al., 2010). These distinct CA-MRSA lineages have arisen almost simultaneously in different regions of the world. Although the pathogen-specific factors contributing to this phenomenon continue to be sought, a common characteristic is that particular host populations have been identified as risk groups for CA-MRSA infections and/or carriage. These populations tend to be socio-economically marginalized with Indigenous, injecting drug user, incarcerated and homeless populations being overrepresented. For example, USA300 infections in the United States were first reported in children in disadvantaged populations in Chicago (Herold et al., 1998), while CA-MRSA in Australia were initially documented from Indigenous populations in Western Australia (Udo et al., 1993). This association is further supported when examining ST80-MRSA emergence in Europe with infections occurring in children from lower socio-economic households (Dufour et al., 2002).

In contrast to healthcare-associated MRSA lineages, which have demonstrated intercontinental transmission with subsequent establishment and spread within local healthcare systems (Harris et al., 2010; Holden et al., 2013), the typical pattern of movement for CA-MRSA lineages appears to be far more geographically restricted. Despite frequent intercontinental traffic between North America and Europe, the dominant USA300 CA-MRSA lineage has not become established in Europe (Toleman et al., 2016; Bouchiat et al., 2017). Similarly, ST80-MRSA in Europe, which likely arose from a methicillin-susceptible *S. aureus* (MSSA) ancestor from sub-Saharan Africa (Stegger et al., 2014), has not become established in the United States. Frequent intercontinental transfers have also been noted for the South-East Asian CA-MRSA clone (ST59), but with limited evidence of ongoing clonal transmission outside the country of origin (Ward et al., 2016).

In Australia, the dominant CA-MRSA lineage is the Panton–Valentine leukocidin positive ST93. First reported in the early 2000s in Queensland on the eastern coast of Australia (Munckhof et al., 2003), ST93-MRSA has rapidly become the most common circulating CA-MRSA lineage (Coombs et al., 2016). Severe clinical manifestations have been widely reported (Peleg et al., 2005; Tong et al., 2008). Murine models suggest ST93 to be more virulent than other CA-MRSA clones including USA300 (Chua et al., 2011; Tong et al., 2013). Previous investigations of ST93 genomics by Stinear et al. (2014) demonstrated limited genetic diversity of the clone, with acquisition of staphylococcal cassette chromosome *mec* element (SCC*mec*) conferring methicillin-resistance on two occasions. Dating and phylogeographical analyses concluded that the most recent common ancestor of ST93 MSSA included in that study arose in the 1970s in North Western Australia. These conclusions were based on a collection of 56 isolates predominantly from Western Australia (representing approximately half of all the isolates). Given these limitations, and the current uncertainty of the ancestry of ST93 relative to other *S. aureus* lineages, we examined the genomics of ST93 on an extended dataset (consisting of an additional 403 ST93 isolates). This collection not only represents a deeper sampling from the hypothesized regions of emergence in Australia, but also a broader sampling from non-Australian regions to encompass the presumed international spread of ST93-MRSA. We describe the relationship of ST93 to other *S. aureus* lineages and impact of recombination on its genome, as well as the emergence of ST93 as a successful MSSA and subsequent CA-MRSA lineage from remote sparsely populated regions of Northern Australia.

## Materials and Methods

### Isolate Collection

Overall 459 sequenced *S. aureus* isolates were included in the study. We set out to capture as much diversity as practicable of ST93, and in so doing, established for whole-genome sequencing a collection of 403 presumed ST93 *S. aureus* (based on local typing schemes) from international locations where ST93 has been reported including Australia, New Zealand, Samoa, and five European countries (**Supplementary Table S1**). All isolates were from patients with invasive and non-invasive infections collected between 1991 and 2012 and confirmed as either MSSA or MRSA. The data were supplemented by sequence reads from a further 56 isolates, sequenced as part of a previous study by Stinear et al. (2014). Metadata details can be found in **Supplementary Table S1**.

### Sequencing and Assembly

Genomic DNA samples were sequenced on the Illumina HiSeq 2500 platform using 151b paired-end TruSeq chemistry. Reads were mapped to the complete JKD6159 reference genome (2,811,435 bp, GenBank: NC017338), an ST93-MRSA-IVa isolated from a patient in Melbourne, Australia in 2004 (Chua et al., 2010) using SMALT. Variants were called using a combination of Samtools mpileup^1^ and bcftools^2^ with filtering for read depth, read base quality, and mapping quality, ensuring only high-quality SNPs were accepted. All variants at either indels or secondary to mobile elements were excluded from the mapping-based analysis.

### The Relationship of ST93 Relative to Other *S. aureus* Lineages

Manually completed genomes of *S. aureus* were obtained from the NCTC3000 reference collection^3^ using nctc-tools^4^ and supplemented with reference sequences from NCBI including the MSHR1132 genome assembly of *Staphylococcus argenteus* (**Supplementary Table S2**). Multi-locus sequence types (MLST) were assigned with *mlst*^5^ and GFF files were generated with a standardized re-annotation of the collection with Prokka (Seemann, 2014). The whole-genome alignment consisted of extracted locally collinear blocks (LCBs) of 500 bp, present in all genomes determined by progressiveMauve (Darling et al., 2010) and ordered against the ST93 reference genome. Recombination in the core genome alignment was detected with Gubbins (Croucher et al., 2015). Pre- and postrecombination maximum-likelihood phylogenies (under the GTR+G model) were generated from the SNP matrices [determined using SNP-sites (Page et al., 2016) and from variant sites determined by Gubbins] employing RAxML-NG v.0.5^6^ with the *S. argenteus* genome MSHR1132 as outgroup and 100 bootstrap replicates. Resultant phylogenies were visualized in Interactive Tree of Life (Letunic and Bork, 2016). The identity and total number of unique sites affected by recombination was computed by taking the set of base pair locations present in all recombinant segments for a lineage. Recombination events were filtered by size and visualized against the respective non-recombinant phylogeny with *plotTree*^7^. To test the accuracy of the results, the aforementioned workflow was repeated using Mugsy instead of progressiveMauve to establish the core genome alignment (Angiuoli and Salzberg, 2011). Similar results were obtained throughout. Nucleotide divergences were computed on the ProgressiveMauve alignment over a non-overlapping 10,000 bps sliding window under the K80 model of evolution in the *dist.dna* function of *ape* for R.

### Phylogenetic Dating

To investigate the temporal evolution of ST93 we used the Bayesian software package BEAST2 (v.2.3) on the 459 ST93 genomes (Bouckaert et al., 2014). A GTR substitution model with gamma correction for among-site variation was implemented using the core genome following masking of homologous recombination identified by Gubbins (Croucher et al., 2015). All combinations of strict, relaxed lognormal, relaxed exponential and random clock models and constant, exponential and skyline population models were evaluated. Models which failed to converge or result in an effective sample size values <200 following 100 million generations, sampling every 1,000 generations, were excluded from further comparison. The best-fit model combination, exponential population with a relaxed lognormal clock model, was determined by marginal likelihoods using the smoothed harmonic mean estimator within the program Tracer v.1.4. A maximum clade credibility tree was created using the treeAnnotator program. Phylogenetic trees were visualized and manipulated with Figtree v1.4.2^8^.

### Phylogenetic Clustering

To determine clusters in the phylogeny, mutual k-nearest neighbor population graphs (mkNNG) were constructed from the cophenetic distances of the BEAST2 phylogeny using package *ape* for R and NetView (Neuditschko et al., 2012; Steinig et al., 2016) with a range of *k* = 1–100. Fast-greedy modularity optimization (Clauset et al., 2004) Walktrap (Pons and Latapy, 2005) and Infomap (Rosvall and Bergstrom, 2010) algorithms were run on each network configuration to define communities in the graph topology. To assess the stability of the communities in the network topology and select an appropriate configuration, the number of detected communities (*n*) was plotted against *k*. The plot indicated a disassociation of the community structure (in line with increasing sparseness of edges) in the network at *k* < 10. Communities found in a conservative and stable configuration of the network (*k* = 60, vertices = 459, edges = 4,711) were selected to determine the general network clusters using the low-resolution fast-greedy modularity optimization algorithm (**Supplementary Figure S7**). Cluster assignments were mapped back to the phylogeny (**Supplementary Figures S7A,B**).

### Global and Regional Transmission Routes

A Bayesian phylogeographic analysis was performed within BEAST2 using the longitude and latitude data from where the sequenced isolate originated (Lemey et al., 2009). Subsequent data were visualized and manipulated in the software tool SPREAD (Bielejec et al., 2011). In addition, ML Ancestral state reconstruction was undertaken to confirm the likely origin of ST93 treating isolate location as a discrete trait in R package *ape*. Skyline population models were examined but excluded as they were found to be biased by non-uniform sampling.

### SCC*mec* Analysis

Alignment of *de novo* contigs to the reference (JKD6159) SCC*mec*IV[2B] revealed two additional SCC*mec* types. Assembly and closure of a novel SCC*mec* type was achieved by mapping contigs to previously described elements and Sanger sequencing to bridge sequence gaps. The variant was referred to the International Working Group on the Classification of Staphylococcal Cassette Chromosome Elements (IWG-SCC) which confirmed the new variant, named SCC*mec*IVn. Two “discordant” isolates (from NZ and Victoria) were detected (i.e., *mecA* negative but phenotype reported as methicillin-resistant). Sequence mapping to SCC*mec* confirmed reads mapping to the components of the cassette suggesting these isolates had lost components of their SCC*mec*, a phenomenon known to occur with storage (van Griethuysen et al., 2005).

### Pan-Genome and Genomic Association

A pan-genome was created from isolate contigs, which were generated with an in-house assembly and improvement pipeline based on Velvet (Zerbino, 2010) and annotated using Prokka (Seemann, 2014). The pan-genome was generated using Roary (Page et al., 2015) and visualized using Phandango (Hadfield et al., 2017).

### Nucleotide Sequence Accession Number

The sequence reads for all isolates are available from ENA (**Supplementary Table S1**).

## Results

### ST93 Is a Divergent and Recombinant *S. aureus* Lineage

**FIGURE 1:**
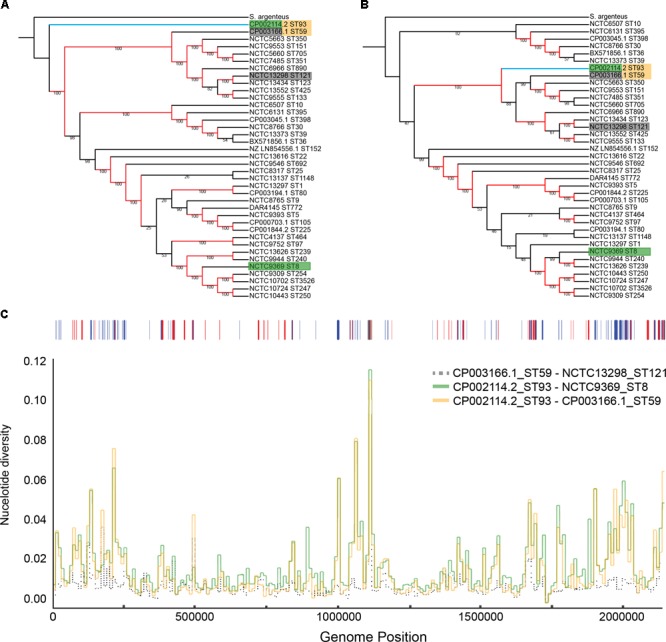
Phylogenetic position of ST93 in relation to other *Staphylococcus aureus*. Maximum-likelihood cladograms (RAxML, GTR+G) generated from complete reference genomes from a core alignment with progressiveMauve before **(A)** and after **(B)** removing recombination with Gubbins. Red branches indicate branches with bootstrap support of 100 and indicate strong support for an early divergence of ST93 (indicated by the blue branch) outside the main group of *S. aureus* sequence types **(A)**. Following masking of recombination, ST93 is located within the larger *S. aureus* complex basal to a larger clade containing other regional clones like ST59 and ST121 **(B)**. Colored boxes around tip labels indicate isolate pairs used in subsequent comparisons. **(C)** Pair-wise nucleotide divergence across a 10,000 bp sliding window along the *S. aureus* core genome with comparisons between ST59-ST121, ST93-ST59, and ST93-ST8. Bases affected by recombination as detected by Gubbins across the ST93 genome are represented above with shared ancestral events (red) and events unique to the ST93 terminal branch (blue). Terminal branch recombination regions align with genomic positions where ST93 is highly diverged compared to other *S. aureus*.

The position of ST93 within the *S. aureus* population structure has never been clearly defined. Indications from multilocus sequence typing loci are that ST93 diverged early from other *S. aureus* but was not ancestral to ST121 or ST59 (Tong et al., 2010). In contrast, when using the entire shared core genome, a maximum likelihood phylogeny places ST93 as the earliest diverging lineage from all other *S. aureus* genomes included in this study, with a bootstrap support of 100% (**Figure 1A**). However, after masking of putative recombined regions in the core genome (21.6% in ST93), ST93 was no longer the earliest diverging *S. aureus* lineage. Instead, it was placed within the broader clade containing ST59 and ST121 (**Figure 1B**). We examined the putative recombinant regions and found no evidence for a large recombined region event, unlike ST239 which has a single chromosomal replacement of ∼600 kb (Robinson and Enright, 2004), but rather evidence for numerous short segments with an average length of 2,209 bp (**Supplementary Figures S1, S2**). More than half of the recombinant regions were unique to the ST93 branch and not shared with other lineages (**Supplementary Figures S1, S2B**). Such terminal recombination events were more common and their contribution to nucleotide variation greater in ST93 compared to other *S. aureus* genomes [as measured by the recombination/mutation ratio (r/m) of 1.96 and the number of recombination events to point mutation ratio (ρ/𝜃) of 0.0167; **Supplementary Figures S2C,D**]. When we examined pair-wise nucleotide divergence of the core genome for the *S. aureus* species we found that for some regions ST93 was equally and highly diverged from the two main branches of *S. aureus* as represented by ST59 and ST8 strains (**Figure 1C** and **Supplementary Figure S3**). The genomic location of these divergent regions correlated with the regions of terminal recombination events detected by Gubbins and included several complete coding regions (**Supplementary Table S3**). For other regions, the pairwise ST93↔ST59 distance was considerably less than the ST93↔ST8 distance and was similar to that of ST59↔ST121. Thus, ST93 has been heavily impacted by recombination, with large parts of its genome showing a specific relationship with the ST59/ST121 group, and other parts descended from *S. aureus* lineages earlier diverging than any of the genomes in this study.

### ST93 Emerged From North-Western Australia Within the Last 50 Years

**FIGURE 2:**
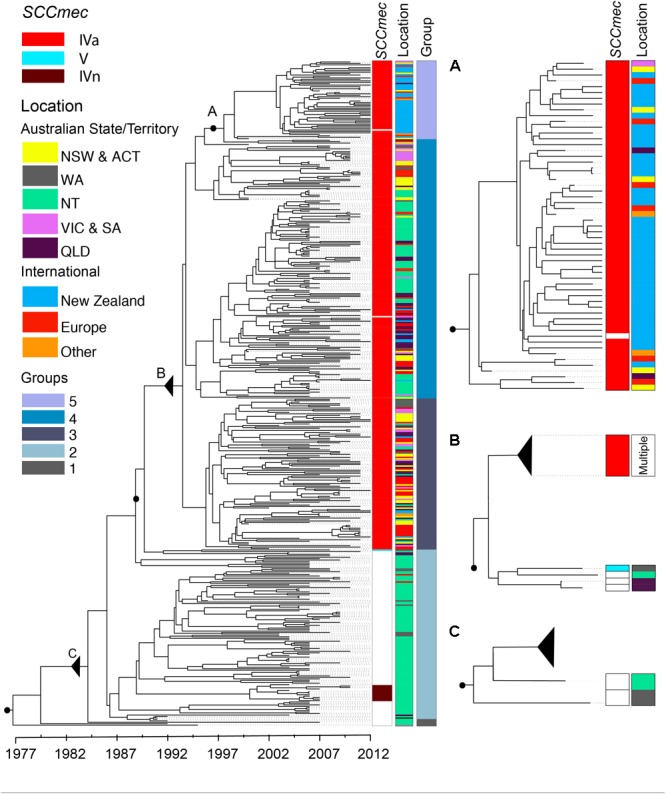
ST93 phylogeny. Maximum clade credibility tree obtained from the core genome not affected by recombination of 459 ST93 sequences using BEAST2. *x*-Axis depicts time in years. Metadata from left to right indicate the presence or absence of the *mecA* gene by SCC*mec* type (i.e., methicillin resistance; white – absence, red – SCC*mec*IVa, brown – SCC*mec*IVn, light blue – SCC*mec*V), the region of origin, and clustering based on the network analysis. Three regions of interest are magnified to the right of the tree with associated metadata. **(A)** Corresponds with group 5 containing predominantly isolates from New Zealand (see text for details). **(B)** Acquisition events of SCC*mec*IVa and SCC*mec*V elements leading to the establishment of ST93-MRSA-IVa. **(C)** The likely origin of ST93 showing the earliest basal strains of ST93-MSSA leading into the main MSSA group. Letters, black filled circles, and solid triangles (collapse point of the rest of the tree) allow for correlation of inserts with the total phylogeny. NZ, New Zealand; EU, Europe; NSW, New South Wales; NT, Northern Territory; QLD, Queensland; VIC, Victoria; WA, Western Australia. Australian Capital Territory (*n* = 1) and South Australian (*n* = 2) isolates are included with NSW and Victorian isolates, respectively.

We determined the phylogenetic structure of a geographically diverse collection of 459 ST93 isolates, which included MSSA (*n* = 112) and MRSA (*n* = 347) (**Figure 2**). The isolates represent an unbiased sampling of *S. aureus* recovered from community and hospital onset skin infections in the Northern Territory (McDonald et al., 2006; Tong et al., 2010), MRSA strains from hospitals and reference collections from Australia, and MRSA strains from public health collections in Europe (mainly the United Kingdom) and New Zealand (**Supplementary Table S1** and **Supplementary Figure S4**). There were 7,044 core genome SNP-sites among the 459 isolates.

**FIGURE 3:**
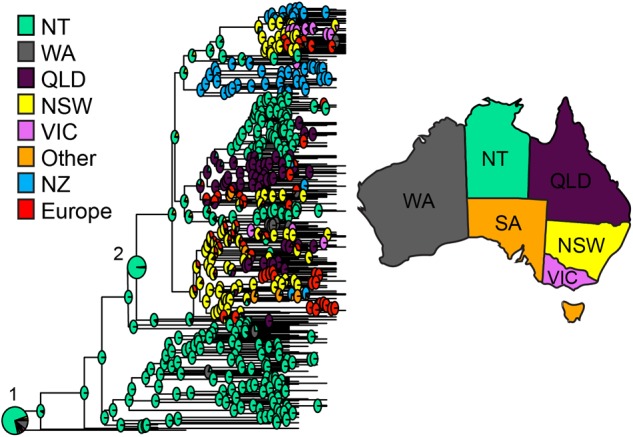
ST93 geographic origins. Ancestral state reconstruction based on maximum clade credibility tree generated using BEAST2 with geographic regions as discrete traits. Each node indicates the log likelihood for specified locations as the origin of the node in pie chart format. Pie charts corresponding with the origins of (1) MSSA and (2) MRSA are enlarged for easier visualization. Abbreviations and colors for regions are as per **Figure 2**.

Dating of the root of the tree (**Figure 2**) suggests the most recent common ancestor of currently extant ST93 isolates to be 1977 [95% highest posterior density (HPD) 1973–1981; and a mutation rate of 1.05 × 10^-6^ (95% HPD 9.8 × 10^-7^ to 1.12 × 10^-6^)]. The ST93-MSSA isolates form a clear basal group, with the vast majority of MSSA isolates (*n* = 104/112), including the most basal MSSA isolates, originating from the bordering jurisdictions of the Northern Territory and Western Australia (**Figure 2C**). Ancestral state reconstruction strongly supports an origin of ST93-MSSA from North Western Australia (**Figure 3**) with the probability heavily weighted toward a Northern Territory origin rather than Western Australia.

### Methicillin Resistance Emerged in ST93 on At Least Three Occasions

**FIGURE 4:**
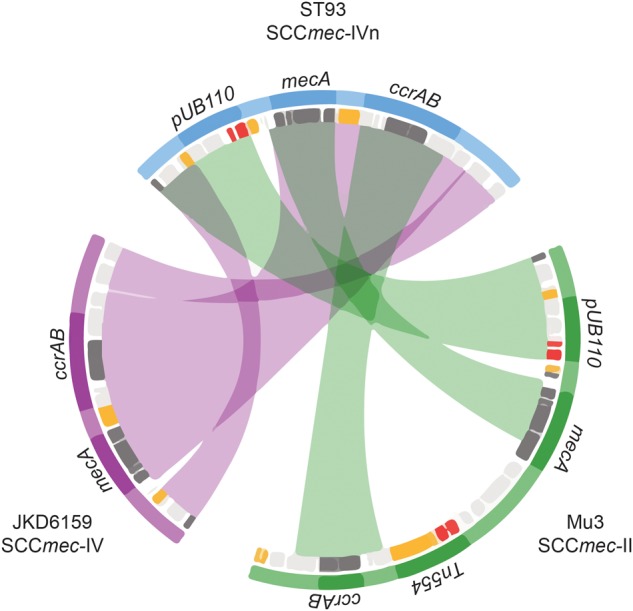
Novel SCC*mec*IVn. Sequence comparison of regions of homology between SCC*mec*II and IV elements from isolates Mu3 (green), ST93 JDK6519 (purple), and the new consensus SCC*mec*IVn from ST93 (blue). Mu3 carries SCC*mec*II and *mec* complex A, JKD6159 SCC*mec*IVa and *mec* complex B, and the novel SCC*mec*IVn is also *mec* complex B. Inner rings denote coding regions (orange – transposition; red – resistance; dark gray – SCC*mec* characteristic regions *orfX, mec* cassette and *ccr* complex; light gray – other). Chords denote major regions (*mec, ccr*, pUB110) defined by nucleotide BLAST comparisons (>99% identity and >1 kb) of SCC*mec*II and IV elements against the SCC*mec*IVn.

Within this diverse MSSA population, SCC*mec* has been acquired on at least three occasions (**Figure 2**). The acquisition of a type IVa SCC*mec* element (SCC*mec*IVa), in the late 1980s or early 1990s, was the basis for a large clonal expansion event, encompassing 332/345 (96.2%) of the MRSA isolates in the study (two isolates were phenotypically MRSA but genotypically lacking *mecA*). A single isolate from Western Australia harbored a type V SCC*mec* element (SCC*mec*V). A separate cluster, consisting of 12 isolates from the Northern Territory was found to harbor a novel SCC*mec* element (GenBank Accession Number KX385846) (**Figure 4**), now designated IVn (SCC*mec*IVn; International Working Group on the Classification of Staphylococcal Cassette Chromosome Elements). The 12 isolates formed a monophyletic cluster, representing a separate and distinct acquisition event, within the Northern Territory. Of the 12 isolates, 11 were *spa* type t202, which is the majority *spa* type in the entire dataset (346/459) and 1 was *spa* type t1811. These two *spa* types differ by the presence of one repeat (t202, 11-17-23-17-16-16-25; t1811, 11-17-17-16-16-25). SCC*mec*IVn is closely related to SCC*mec*IVa of the reference strain JKD6159 (ST93-MRSA-IVa), and shares >98% nucleotide identity apart from a pUB110 insertion flanked by IS*431* elements in the J3 region of SCC*mec*IVn (**Figure 4**). The pUB110 element displays >99% identity to pUB110 in the type II SCC*mec* elements (SCC*mec*II) from Mu3, Mu50, MRSA252, and JKD6008, and contains an *ant4-1/aadD* aminoglycoside resistance gene that has been described to confer high-level resistance to tobramycin, amikacin, and kanamycin, as well as the bleomycin resistance gene, *bleO*.

The geographical origins of the ST93-MRSA-IVa lineage have to date remained unclear. ST93-MRSA-IVa was first described in South East Queensland in 2000 as a cause of skin and soft tissue infections (SSTI) in mainly Caucasian patients (Munckhof et al., 2003), and then in 2003 as a cause of severe disease in mostly Indigenous Australian patients (Peleg et al., 2005). In our dataset, the earliest ST93-MRSA-IVa strains were isolated in 2000, and eight of these nine strains, were from the eastern states of Australia (New South Wales/Australian Capital Territory: SAPWH23, SAPWH39, SAPRPAH96, SAPTCH92, SAPWH61, SAPWH64; Queensland: SAPRBH98; Victoria: SAPRCH74; and South Australia: SAPGPSA73). Notably, despite comprehensive state-wide typing of MRSA isolates since 1996 in Western Australia, ST93-MRSA was not identified in Western Australia until 2003. Ancestral state reconstruction (**Figure 3**) indicates that the ST93-MRSA-IVa lineage most likely emerged from the Northern Territory in the early 1990s. By the year 2000, transmission from the Northern Territory to the eastern states of Queensland and New South Wales had occurred.

### International Transmission of ST93-MRSA-IVa

Network clustering delineated five main ST93 groups: Groups 1 and 2 representing MSSA strains and groups 3, 4, and 5 representing the three major groups within the ST93-MRSA-IVa clade (**Figure 2**). Group 3 is principally comprised of strains from the Australian east coast (81/105 strains are from New South Wales, Australian Capital Territory, Victoria, South Australia, and Queensland) and Europe.

European isolates (mainly from the United Kingdom) are highly diverse and are scattered across groups 3, 4, and 5 consistent with multiple sporadic exportations to the United Kingdom (and possibly elsewhere in Europe). Despite frequent exportation events and a limited number of transmission chains within the United Kingdom [**Supplementary Figure S5**; with one cluster of three isolates originating from the same pediatric hospital (**Supplementary Figure S5** – Box 2) and one cluster of four isolates recovered from an index case and siblings in the same household (**Supplementary Figure S5** – Box 1)], no evidence for sustained transmission in the United Kingdom was seen.

**FIGURE 5:**
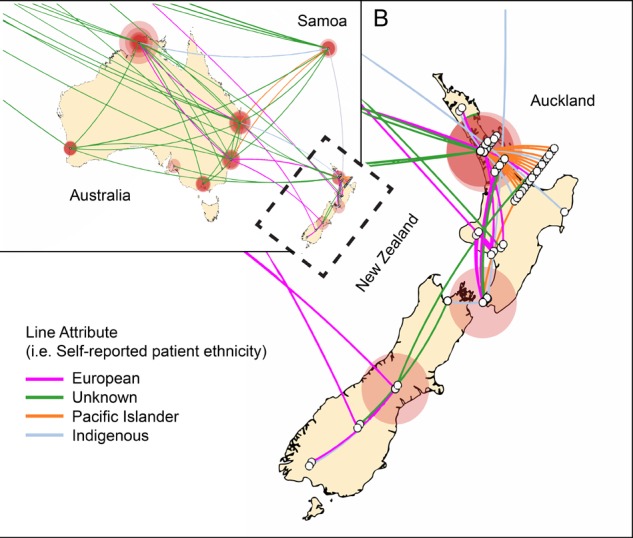
Transmission of ST93-MRSA-IVa in Australia and New Zealand. Phylogenetic diffusion of ST93 within New Zealand based on discrete traits implemented using SpreaD3. Oceania region is depicted with the outlined area (New Zealand) enlarged in insert B. White open circles represent the origin of the isolate. For isolates from the same location a jiggle factor is included such that intra-local diffusion is visible. Connecting colored lines represent the ethnicity of the host as shown in the legend. Circular polygons are proportional to the number of tree lineages maintaining that location. Indigenous ethnicity reflects either an Aboriginal Torres Strait Islander (Australian) or Maori (New Zealand) host. The analysis shows that within New Zealand maintenance of ST93 is centered on Auckland within the Maori and Pacific Islander communities and that introductions of this clone are linked to travel between New Zealand and Australia.

In contrast to the epidemiological picture in Europe, there was evidence for sustained transmission and endemic spread of ST93-MRSA-IVa in New Zealand. The majority of New Zealand isolates cluster together within group 5 (**Figure 2**). This strongly suggests establishment in around 2000 followed by clonal expansion and sustained transmission of a New Zealand specific ST93-MRSA-IVa lineage. In addition, not only is there evidence of ongoing transmission in New Zealand, but also instances of transmission back to Australia and to Europe. Given the novelty of this observation with respect to CA-MRSA lineages (i.e., introduction and establishment of a CA-MRSA lineage), we examined the epidemiological characteristics associated with these New Zealand isolates. Significantly, 52% (28/54) of all ST93-MRSA-IVa from New Zealand [or 63% (25/40) of group 5] were centered around Auckland on the north island and principally originating from within the Māori and Pacific Islander populations [of New Zealand isolates with a known host ethnicity, 32/49 (65%) of ST93-MRSA-IVa overall, and 24/38 (63%) of group 5, were recovered from Māori and Pacific Islanders]. A Spatial Phylogenetic reconstruction (**Figure 5**) supported transmissions between Australia and New Zealand via hosts of European or unknown ethnicity, while the majority of within New Zealand transmission was within the Pacific Islander population.

The contrast between the epidemiology in the United Kingdom and New Zealand is further supported by the longitudinal data (**Supplementary Figure S6**) based on contemporary MRSA typing results (between 2008 and 2015) from the United Kingdom, New Zealand and Australia, which indicate consistently low levels of ST93 in the United Kingdom and therefore likely ongoing sporadic importation, compared to continued increases in Australia and New Zealand consistent with sustained transmission and endemic spread within these two countries.

### Genomic Markers of Specific Clades

We examined the dataset for genetic markers that may provide clues to the success of specific clades within the overall ST93 population. Twelve single nucleotide variants (SNVs) mapped to the branch corresponding to SCC*mec*IVa acquisition, of which 11 SNVs were not associated with known virulence genes. One SNV introduced a premature stop codon in *aryK*, a novel virulence regulator as previously described by Chua et al. (2014). It has been hypothesized that this loss-of-function point mutation may act to reduce the virulence of ST93 and contribute to host persistence. However, the ongoing circulation of ST93-MSSA in the Northern Territory suggests that ST93 is quite capable of successful persistence despite the lack of an SCC*mec* element and the absence of this loss-of-function point mutation.

We also looked at variants restricted to the New Zealand clade (group 5) (**Supplementary Table S4**). Fourteen SNVs were found of which eight were non-synonymous. Of note, a mutation in *adsA* (also known as *sasH*), encoding a surface bound protein adenosine synthase, was detected. *AdsA* is a critical virulence factor in *S. aureus* and converts adenosine tri-, di-, and monophosphate to adenosine, facilitating escape from phagocytic clearance (Lemey et al., 2009; Bielejec et al., 2011). However, the P33S substitution conferred by the mutation sits outside both the metallophosphatase and 5′-nucleotidase conserved domains of *adsA*, and thus the functional implications of this mutation with regards to fitness or virulence are unknown, and will require further laboratory experiments to elucidate.

The overall ST93 gene content (pan-genome) consisted of 3,837 genes, of which the core genome (present in over 99% of isolates) comprised 2,436 genes. Of the remaining 1,401 genes outside of the core genome, 1,212 were found in <15% of isolates, consistent with a significant flux of accessory genes. Apart from genes associated with SCC*mec* elements, we did not find associations between particular accessory genome elements and the major clades. 455/459 isolates harbored the PVL genes *lukS-PV* and *lukF-PV*. The Roary output for the pan-genome can be found in **Supplementary Table S5**.

## Discussion

The evolutionary history of ST93 represents the emergence from isolated and sparsely populated regions in Australia of a virulent community-associated *S. aureus* lineage, which has become established more broadly in Australia. Global transmission has also occurred in different patterns, with sporadic transmission to the United Kingdom and elsewhere in Europe and contrastingly, sustained endemicity in the New Zealand Pacific Island population. These findings suggest that as much as pathogen characteristics are important, aspects of the host population play a significant role in the success of bacterial lineages.

At a whole genome level, ST93 can be considered the earliest diverging of currently sampled *S. aureus* lineages. We found evidence that recombination has played a significant role in the evolutionary history of ST93 with large proportions of the genome derived from highly divergent sequences and other segments from ancestors related to the ST59/ST121 clade of *S. aureus*. Somewhat at odds with this ancient history is that the currently extant ST93 population has limited genetic diversity with a most recent common ancestor from the 1970s.

The current ST93 population has rapidly become established in geographically disparate remote regions of the Northern Territory and Western Australia with largely Indigenous Australian populations. In a survey of Indigenous Australian residents from remote communities in northern Western Australia from 1996 to 2003, the most common lineage isolated was ST93-MSSA (O’Brien et al., 2009). Broader epidemiological studies with an unbiased sampling of both MSSA and MRSA in Australia concur with a substantial presence of ST93-MSSA in the Northern Territory (Tong et al., 2010) and Western Australia (O’Brien et al., 2009) and its near absence in other parts of Australia (Holmes et al., 2014). A possible interpretation of our results is that ST93, with genomic regions derived from an early diverging population of *S. aureus*, has been carried in isolation by Indigenous Australians, also an early diverging population within *Homo sapiens* (Malaspinas et al., 2016). However, the dating of the emergence of ST93 to the 1970s is inconsistent with this model unless there was a tight bottleneck in the 1970s leading to a marked reduction in diversity of ST93. An alternative scenario is that the expansion of ST93 in humans followed a cross-species transfer event from a yet to be identified non-human reservoir.

The recent expansion and establishment of a *S. aureus* clone may be somewhat surprising given the overall sparseness of population in these regions; the Northern Territory has a population density of 0.2 person per km^2^, and the Kimberley region in the north of Western Australia <0.1 person per km^2^, compared to 2.9 and 269 people per km^2^ for Australia and the United Kingdom, respectively. Notably, the Northern Territory and Kimberley, Western Australia have among the highest proportions of Indigenous people as part of the population in Australia at 26% (and 51% if excluding the urban center of Darwin) and 41%, respectively. There is documented overcrowding within remote community households with a median of 7 and up to 23 residents per household (Vino et al., 2017) and a high prevalence of SSTI (an estimated 45% of remote living Indigenous children from the Northern Territory, Western Australia and Queensland suffer from impetigo at any one point in time) (Bowen et al., 2015), facilitating the spread and amplification of local emerging *S. aureus* clones. Studies in the United States and United Kingdom have highlighted the importance of household transmission and markers of socio-economic deprivation to the transmission of CA-MRSA lineages (Knox et al., 2015; Tosas Auguet et al., 2016).

Strikingly, ST93-MRSA-IVa was not detected in Western Australia until 2003. However, since then ST93-MRSA-IVa has become the dominant CA-MRSA in Western Australia, with a focus in the north-west of the state, particularly the Kimberley region where ST93-MRSA has been isolated at a rate of 2,499 isolations per 100,000 population in 2015–2016 (compared to 41 isolations per 100,000 population in the metropolitan region around the capital Perth) (Coombs et al., 2017). As with the emergence of ST93-MSSA, it is likely that socio-demographic factors play an important role in this rapid expansion of ST93-MRSA-IVa.

Following its emergence in north-western Australia, ST93-MRSA-IVa became endemic in Australia, and then was transmitted from the Australian eastern seaboard to the United Kingdom and New Zealand. Similar to patterns seen with USA300 (Bouchiat et al., 2017) and ST80 (Stegger et al., 2014), inter-country transmission has not led to continued transmission and endemicity of ST93-MRSA-IVa in the United Kingdom. In contrast, we have demonstrated establishment of ST93-MRSA-IVa in New Zealand, centered primarily in the Auckland region and in the Pacific Islander population. Māori and Pacific Islanders made up 65% of New Zealand ST93-MRSA-IVa infections and are thus clearly over-represented in light of these population groups representing 15 and 7%, respectively of the general New Zealand population. Notably, rates of *S. aureus* skin infections and CA-MRSA infections are significantly higher in Mâori and Pacific Islander populations in New Zealand compared to patients of European ethnicity (Williamson et al., 2013, 2014). The reasons for this association are multifactorial, but are likely to include a combination of poverty and domestic overcrowding – i.e., similar socio-demographic conditions to those favoring the emergence of ST93 in north-western Australia (Williamson et al., 2013, 2014). These data may explain why other CA-MRSA lineages remain currently geographically restricted, despite numerous intercontinental transfer events, as incursion events are not usually occurring into a vulnerable population.

## Conclusion

ST93 is an early diverging lineage of currently sampled *S. aureus* lineages, with a genome shaped by recombination and of which large proportions are derived from highly divergent sequences. The evolutionary history of ST93 suggests the importance of host population characteristics on both the emergence and ongoing spread of CA-MRSA lineages. Understanding this interplay better would enable interventions at controlling these clones to be refined.

## Data Availability

The sequence reads for all isolates are available from ENA (**Supplementary Table S1**).

## Author Contributions

SH, ES, PG, and ST designed and undertook the analysis. SH, PG, and ST wrote the manuscript. PA conducted aspects of the bioinformatic analyses. SRH and MH provided bioinformatic advice and interpreted the results. SH, PG, DH, and ST designed the study. SB, JP, PG, and ST were responsible for the management of the study. GN, DW, HH, SR, AK, ME, ED, HL, GC, DH, PG, and ST identified and collected isolates and relevant clinical information. All of the authors read, modified, and approved the manuscript.

## Conflict of Interest Statement

JP is a paid consultant at Specific Technologies LLC. The remaining authors declare that the research was conducted in the absence of any commercial or financial relationships that could be construed as a potential conflict of interest. The handling Editor declared a past co-authorship with one of the authors AK.

## Footnotes

1 http://samtools.sourceforge.net

2 https://samtools.github.io/bcftools/

3 http://sanger.ac.uk/resources/downloads/bacteria/nctc/

4 https://github.com/esteinig/nctc-tools

5 https://github.com/tseemann/mlst

6 https://github.com/amkozlov/raxml-ng

7 https://github.com/katholt/plotTree

8 http://tree.bio.ed.ac.uk/software/figtree

## Supplementary Material

FIGURE S1 Recombinant regions across *Staphylococcus aureus* as detected by Gubbins. Maximum-likelihood cladograms (RAxML, GTR+G) of the progressiveMauve (2,157,328 sites, 358,325 SNPs) core alignment after removing recombinant segments (blocks). Recombinant segments were filtered by length as indicated and represent shared ancestral events (red) and events unique to terminal branches (blue). Large segments spanning the origin of replication are present in the ST239/ST240 lineage, while smaller events are particularly common in *S. argenteus* and lineages ST22, ST93, ST152, and ST395.

FIGURE S2 Terminal recombination events. Terminal recombination events (unique to each sequence type) detected with Gubbins in the progressiveMauve core genome alignment (2,157,328 sites). **(A)** Length distribution of unique recombinant events demonstrating a majority of short recombination events (average: 2,209 base pairs) detected in ST93. **(B)** Total base pairs of non-overlapping recombinant events ancestrally shared (red) or unique (blue) across sequence types in our data set. ST93 (top) features the largest number of unique recombinant sites compared to other sequence types. **(C)** Ratio (r/m) of base substitutions predicted to have been imported through recombination (r) to those occurring through point mutations (m), showing only sequence types with unique (terminal) recombination events on their respective branch of the phylogeny inferred with Gubbins. Thus, there is a strong influence of recombination on the variation accumulated in ST93. **(D)** Ratio (rho/theta) of the number of recombination events (rho) to point mutations, showing a comparatively high rate of recombination in the core genome relative to mutation for ST93.

FIGURE S3 Additional pair-wise nucleotide divergence comparisons between representative *S. aureus* genomes including ST93. Pair-wise nucleotide divergence across a 10,000 bp sliding window along the *S. aureus* core genome with comparisons between ST59-ST121, ST93-ST59, ST93-ST8, ST59-ST8, and ST8-ST5. Bases affected by recombination as detected by Gubbins across the ST93 genome are represented above with shared ancestral events (red) and events unique to the ST93 terminal branch (blue). Terminal branch recombination regions align with genomic positions where ST93 is highly diverged compared to other *S. aureus*.

FIGURE S4 Source of isolates. **(A)** Depicts the location from where the isolates originated with red boxes representing states and territories of Australia. European countries include England (*n* = 45), Scotland (*n* = 12), Denmark (*n* = 4), France (*n* = 1), and Italy (*n* = 1). Note, Samoan isolates (*n* = 4) are not depicted. The number of MSSA isolates sequenced, by location are shown on the secondary axis by the blue line. **(B)** Represents the temporal distribution of the isolates with the number of blood stream isolates depicted by the red bars on the secondary axis. Note that data on source of infection was unknown for 52 isolates.

FIGURE S5 Evidence of short transmission chains in the United Kingdom. Maximum clade credibility tree as in **Figure 1**, but with transmission chains from the United Kingdom highlighted. Box 1 – four isolates from three individuals of which two were siblings. Box 2 – three isolates from separate patients in same pediatric hospital. Box 3 – there were no obvious epidemiological links with cases geographically widely distributed. Box 4 – there were no obvious epidemiological links.

FIGURE S6 Proportion of methicillin-resistant *Staphylococcus aureus* that are ST93-MRSA. This represents longitudinal data of ongoing MRSA typing data from Australia, New Zealand (NZ) and United Kingdom (UK). Graph depicts the percentage of MRSA isolates that are ST93 between 2008 and 2015. Data were obtained from Public Health England, United Kingdom, the Institute of Environmental Science and Research, New Zealand, and the Australian Collaborating Centre for Enterococcus and *Staphylococcus* Species (ACCESS) Typing and Research, Australia.

FIGURE S7 Network clusters of ST93. **(A)** General network clusters using the low-resolution fast-greedy modularity optimization algorithm. **(B)** Cluster assignments were mapped back to the phylogeny.

TABLE S1 Details of 459 ST93 *Staphylococcus aureus* genomes.

TABLE S2 Details of *Staphylococcus aureus* genomes used to determine phylogenetic position of ST93.

TABLE S3 Coding sequences in highly divergent regions of ST93. The divergence peaks in **Figure 1** were determined using a sliding window of 10,000 bp to assess divergence between the pairwise combinations of sequence types. These annotated genes are extracted from each plateau (i.e., 10,000 bp of divergent peak) and from pairwise combinations involving ST93 where >0.05 divergence was noted.

TABLE S4 Non-synonymous mutations restricted to the branch defining the New Zealand clade.

TABLE S5 Pan-genome details from Roary output.
