# Supplementary figures and images for: Global Scale Dissemination of ST93: A Divergent Staphylococcus aureus Epidemic Lineage That Has Recently Emerged From Remote Northern Australia
Source: Front Microbiol. 2018 Jul 9;9:1453. doi: 10.3389/fmicb.2018.01453 (PMC6047344; doi:10.3389/fmicb.2018.01453)

> 0 bp

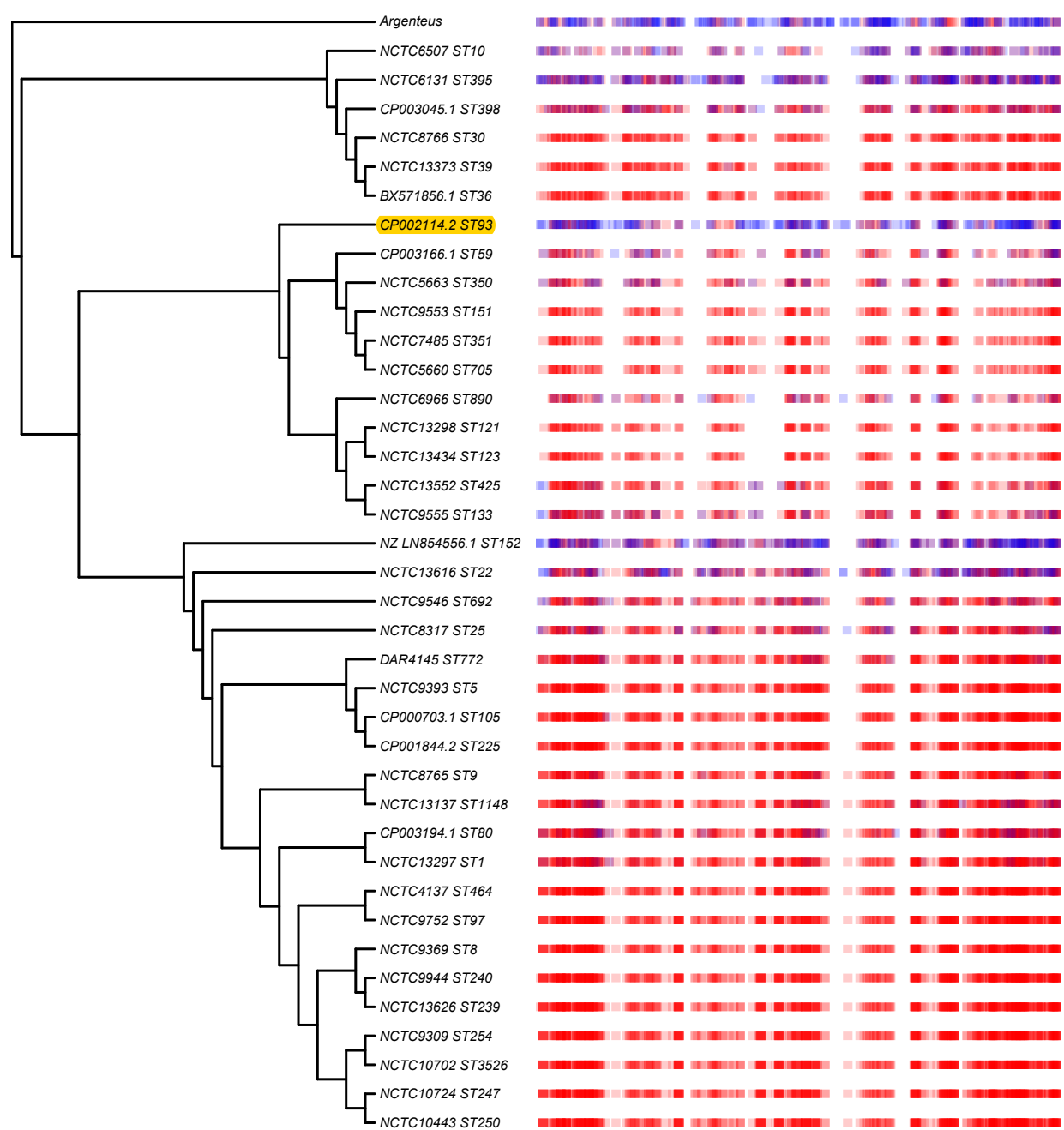

> 500 bp

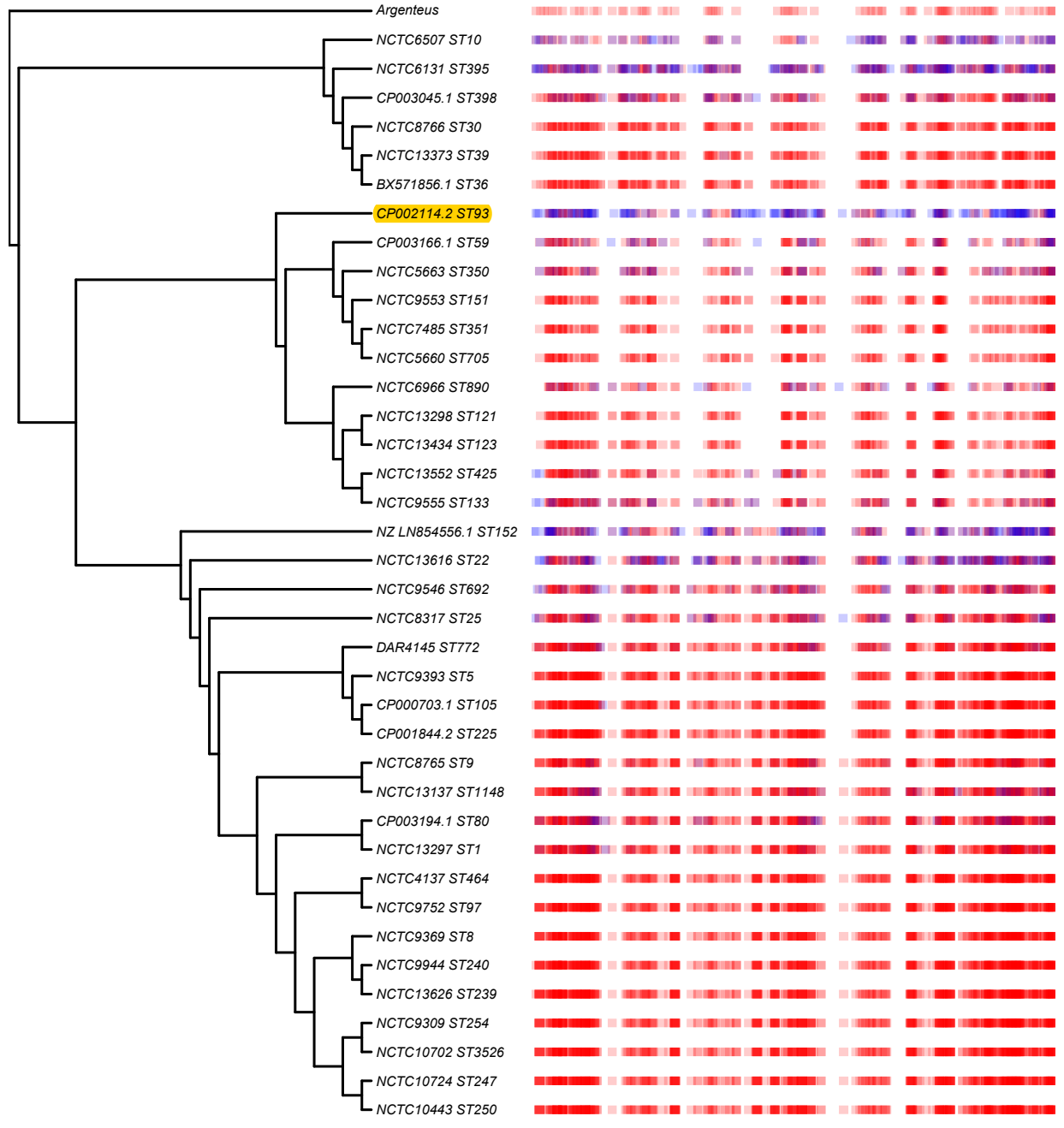

> 1000 bp

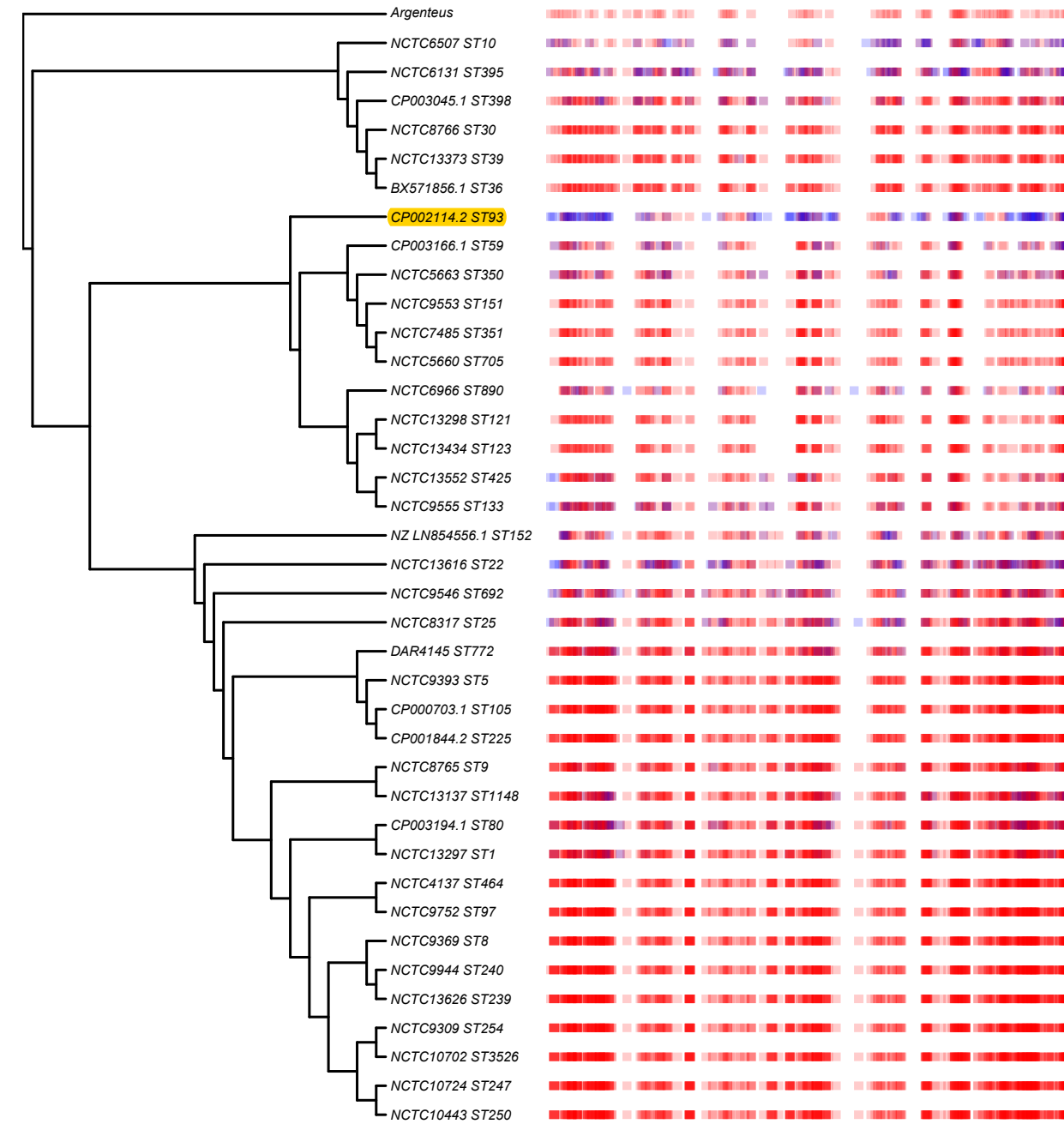

> 5000 bp

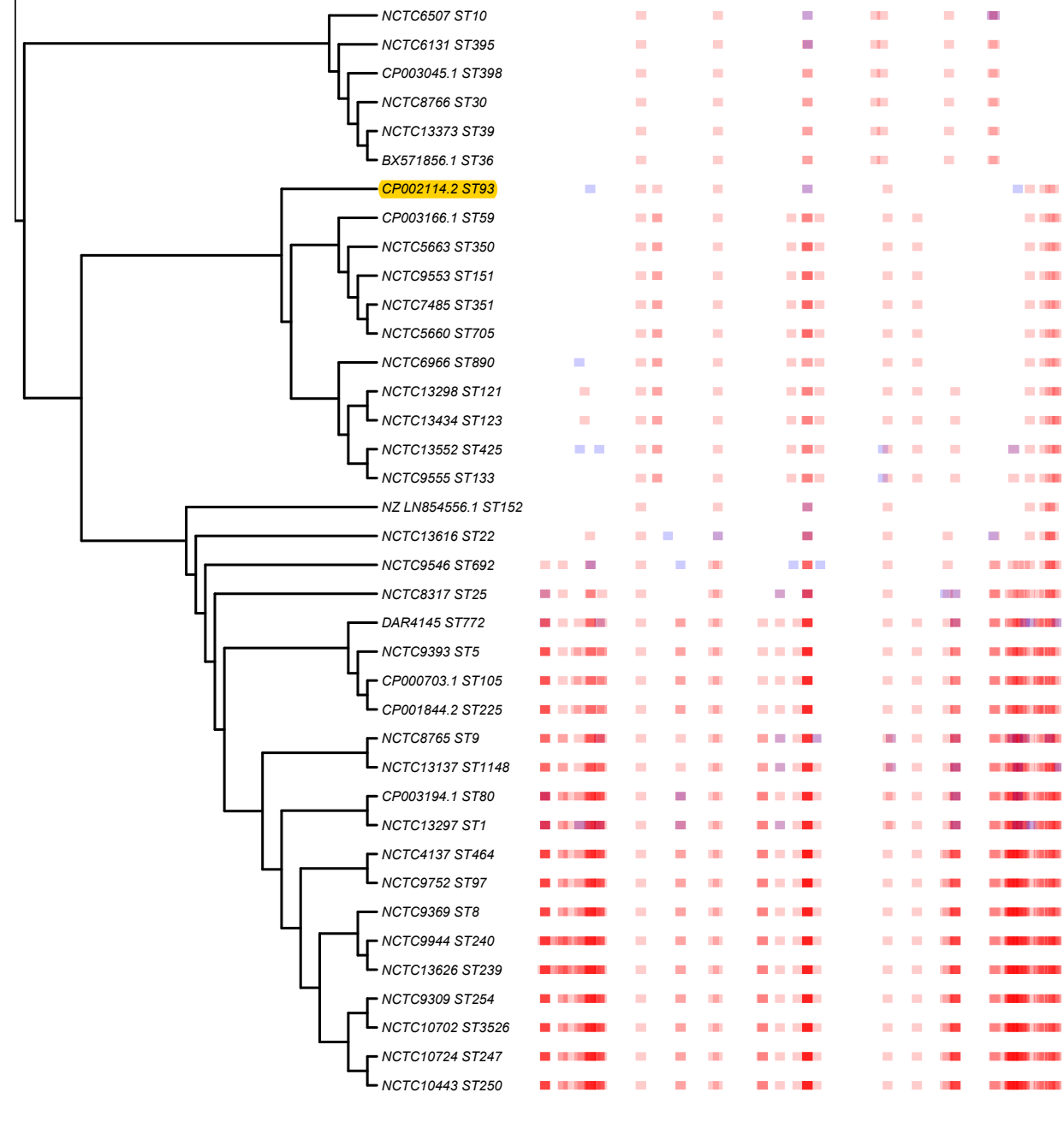

> 15000 bp

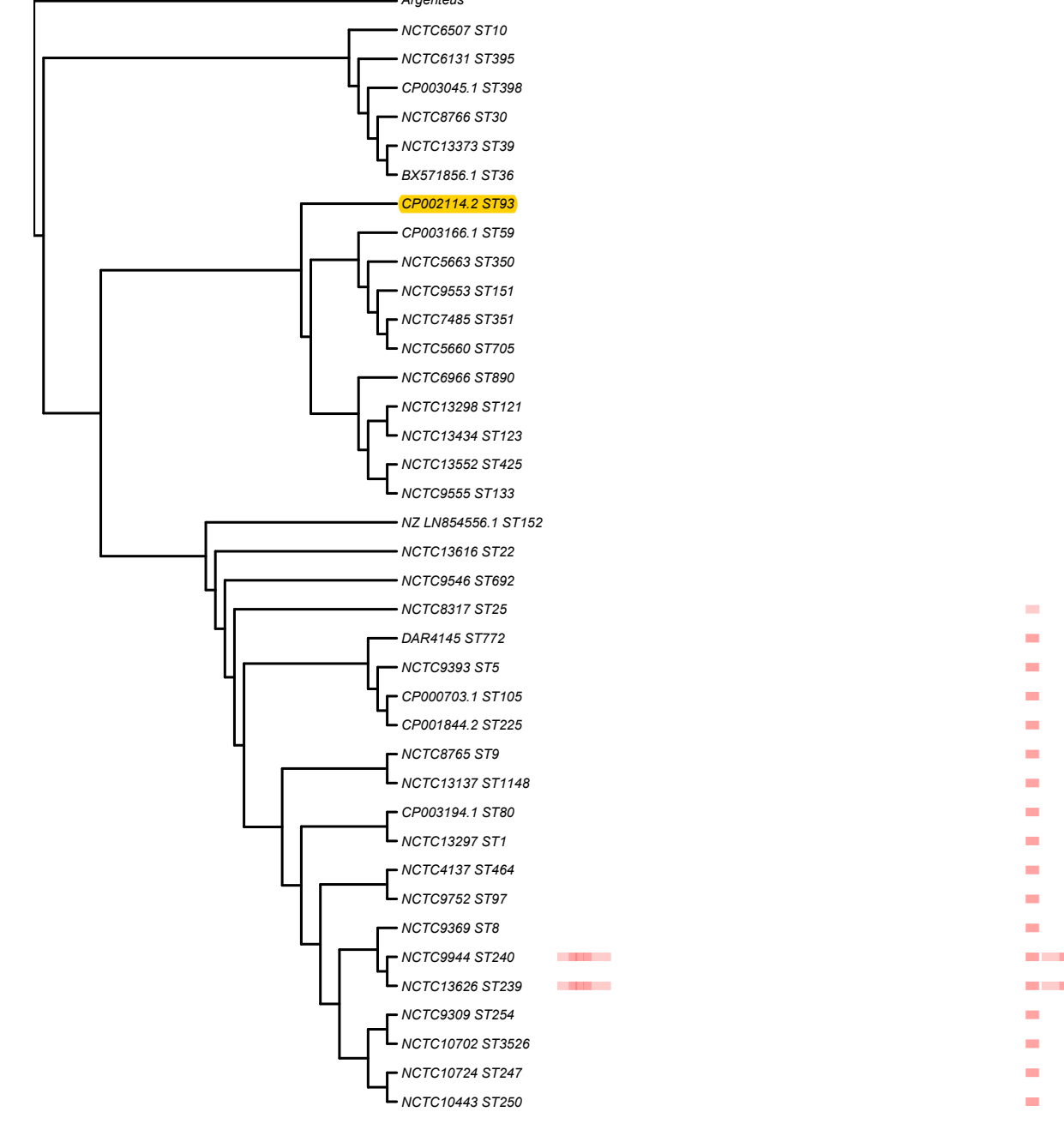

Supplement: FIGURE S1 — Recombinant regions across Staphylococcus aureus as detected by Gubbins. Maximum-likelihood cladograms (RAxML, GTR+G) of the progressiveMauve (2,157,328 sites, 358,325 SNPs) core alignment after removing recombinant segments (blocks). Recombinant segments were filtered by length as indicated and represent shared ancestral events (red) and events unique to terminal branches (blue). Large segments spanning the origin of replication are present in the ST239/ST240 lineage, while smaller events are particularly common in S. argenteus and lineages ST22, ST93, ST152, and ST395. [file Image_1.PDF]

# A

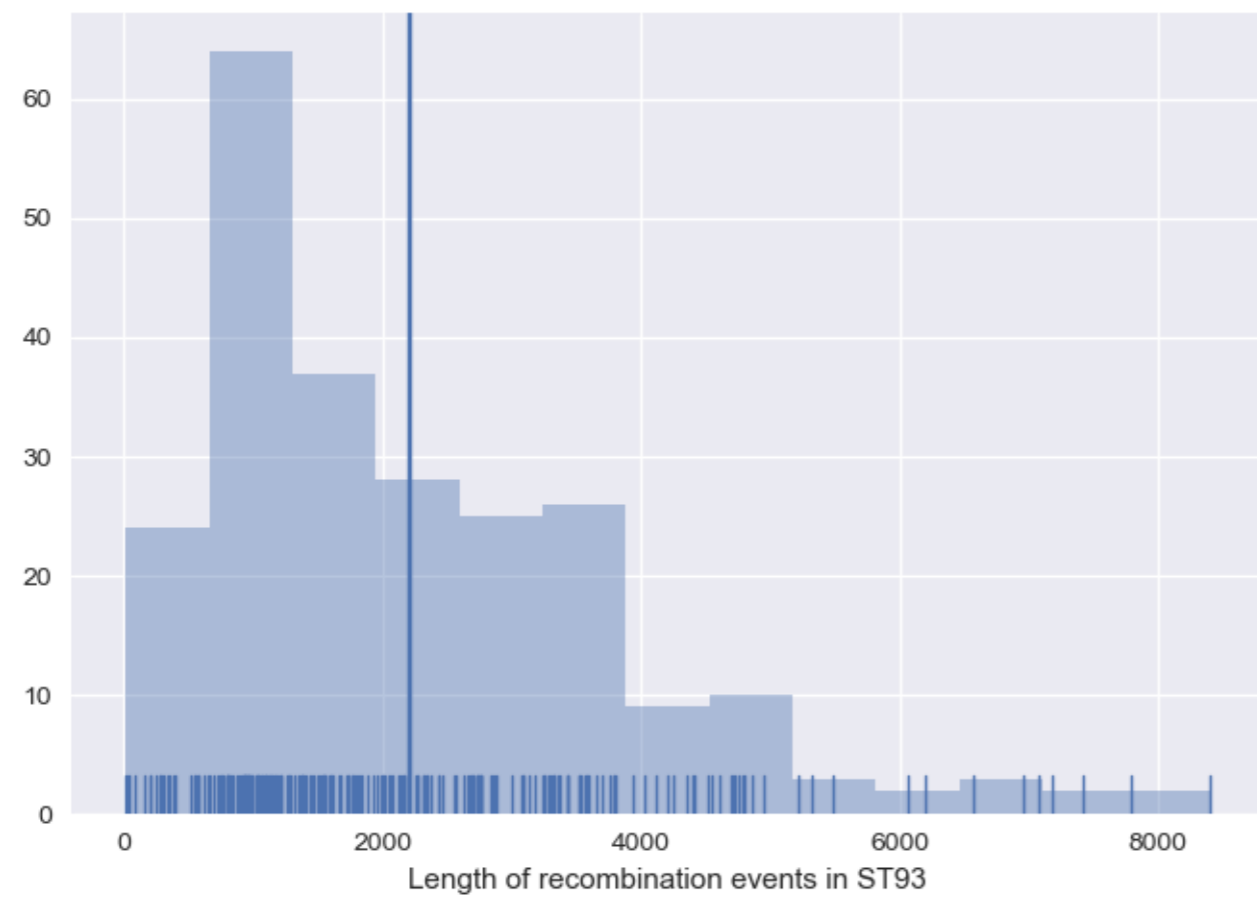

# B

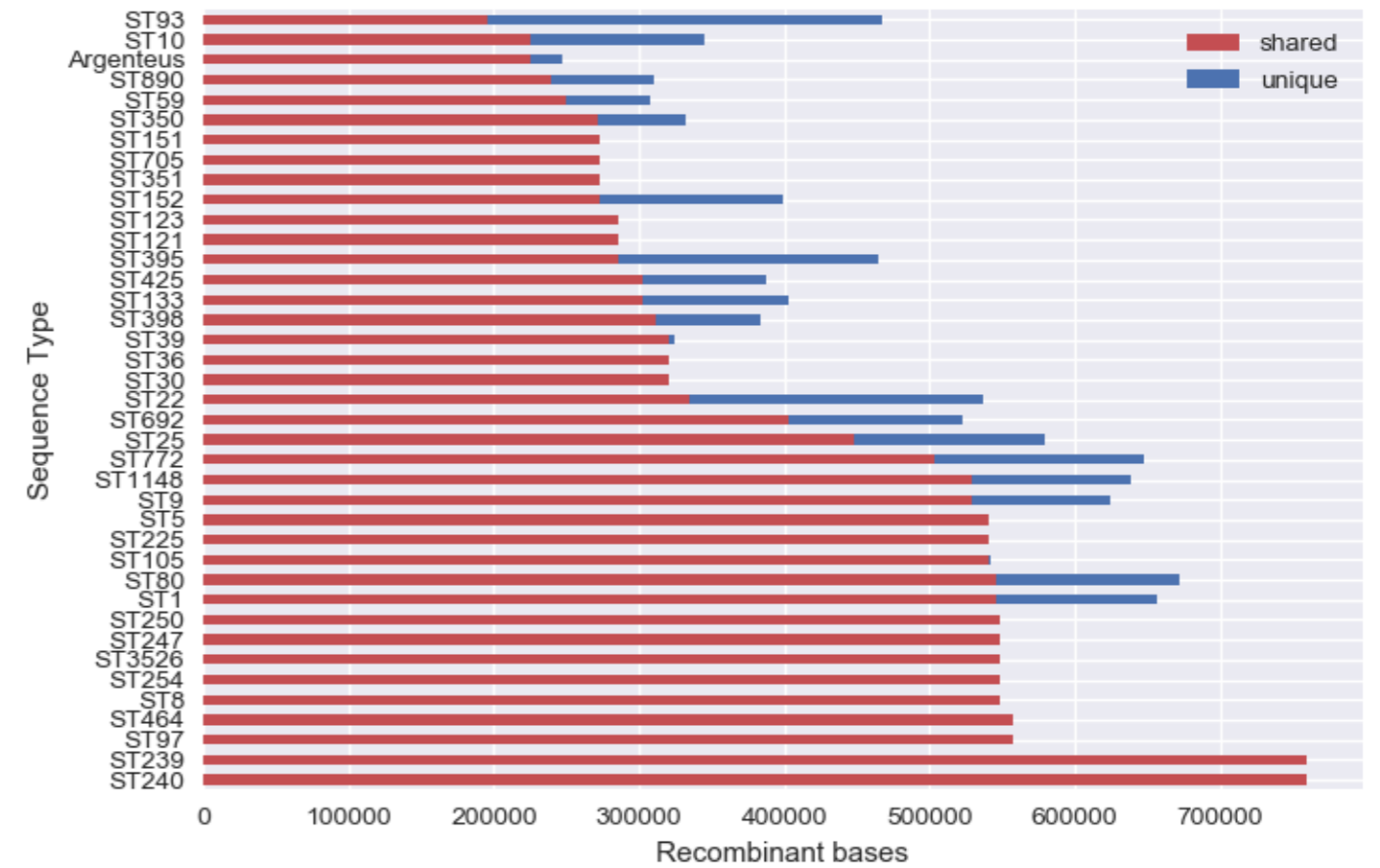

# C

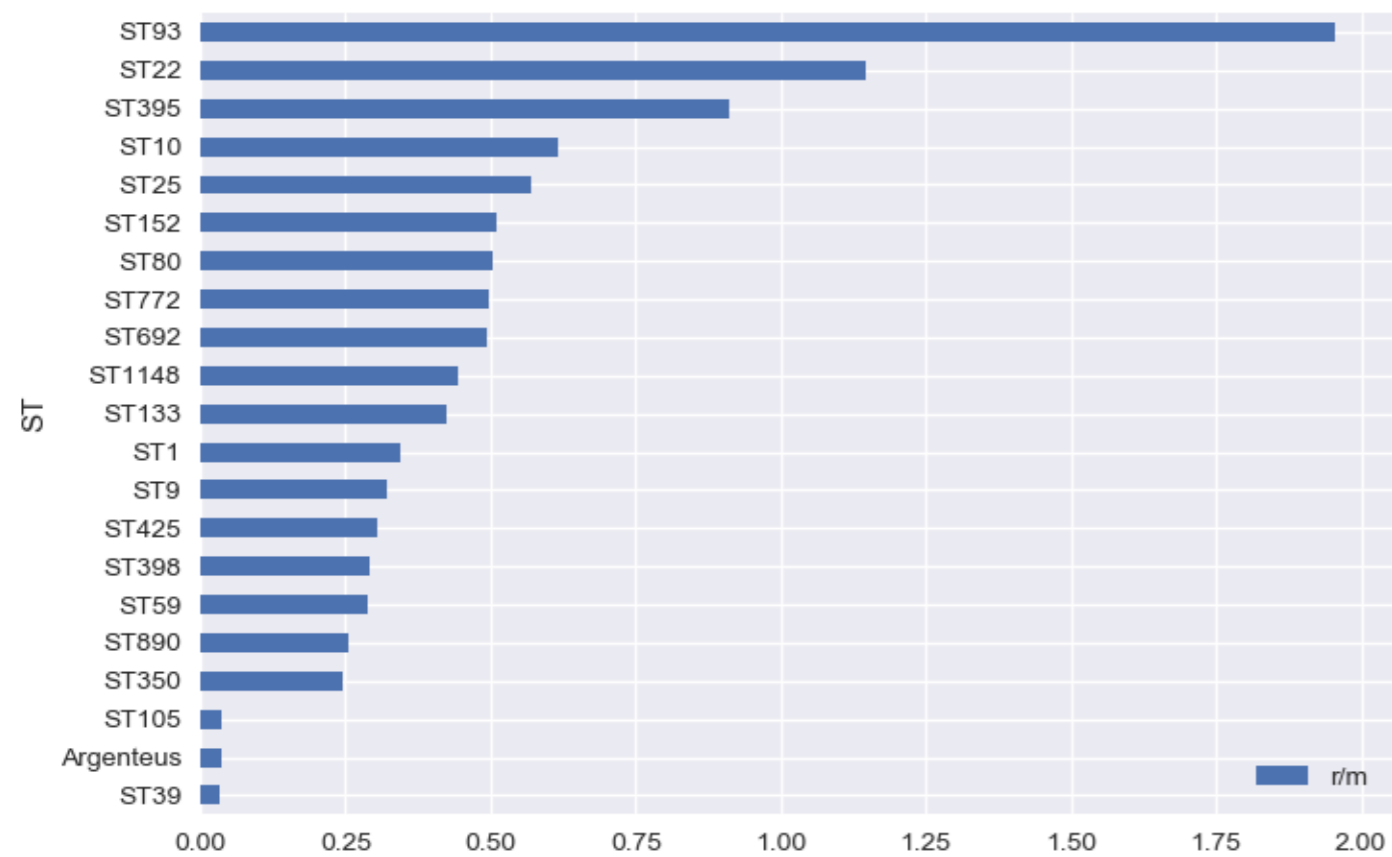

# D

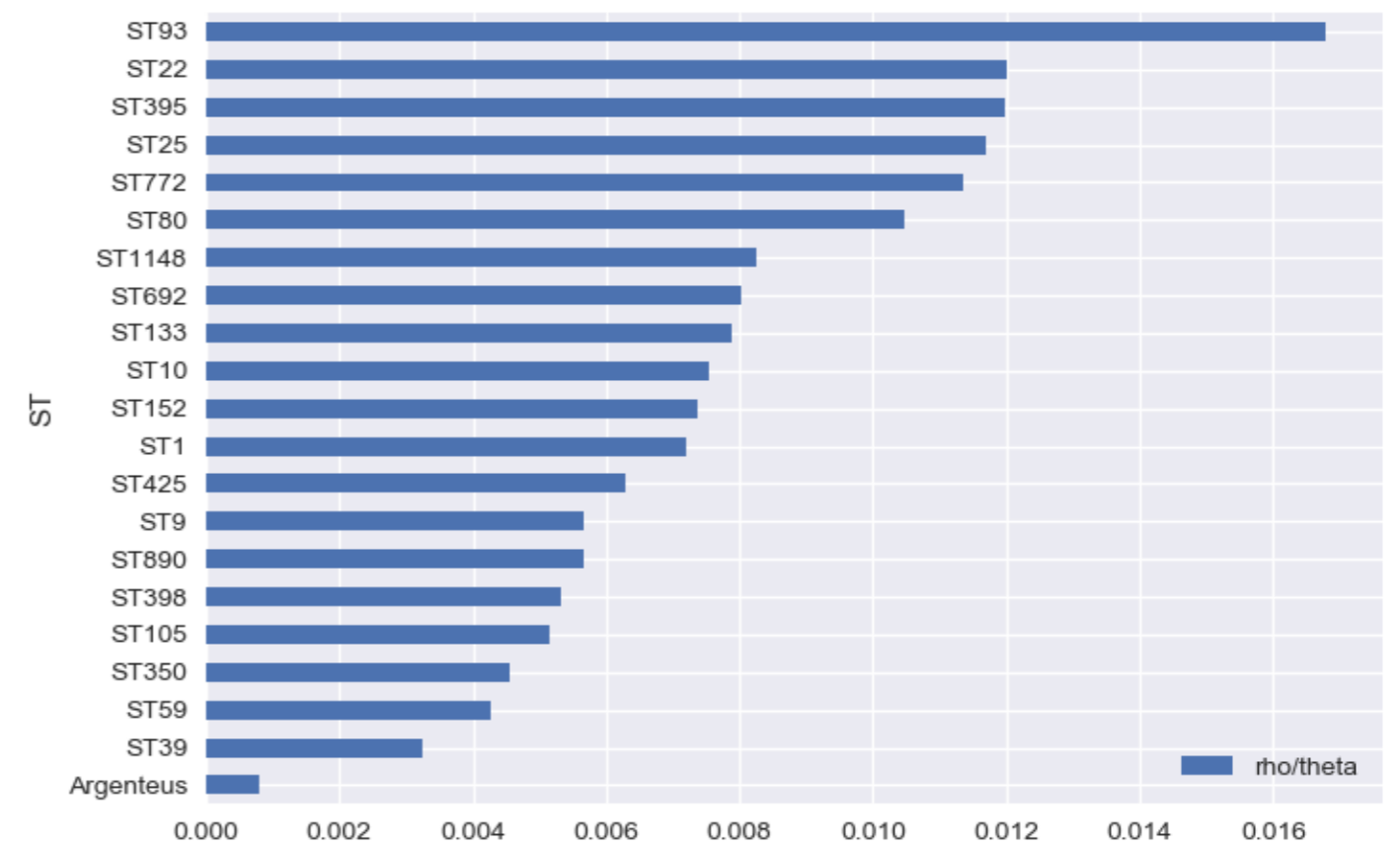

Supplement: FIGURE S2 — Terminal recombination events. Terminal recombination events (unique to each sequence type) detected with Gubbins in the progressiveMauve core genome alignment (2,157,328 sites). (A) Length distribution of unique recombinant events demonstrating a majority of short recombination events (average: 2,209 base pairs) detected in ST93. (B) Total base pairs of non-overlapping recombinant events ancestrally shared (red) or unique (blue) across sequence types in our data set. ST93 (top) features the largest number of unique recombinant sites compared to other sequence types. (C) Ratio (r/m) of base substitutions predicted to have been imported through recombination (r) to those occurring through point mutations (m), showing only sequence types with unique (terminal) recombination events on their respective branch of the phylogeny inferred with Gubbins. Thus, there is a strong influence of recombination on the variation accumulated in ST93. (D) Ratio (rho/theta) of the number of recombination events (rho) to point mutations, showing a comparatively high rate of recombination in the core genome relative to mutation for ST93. [file Image_2.PDF]

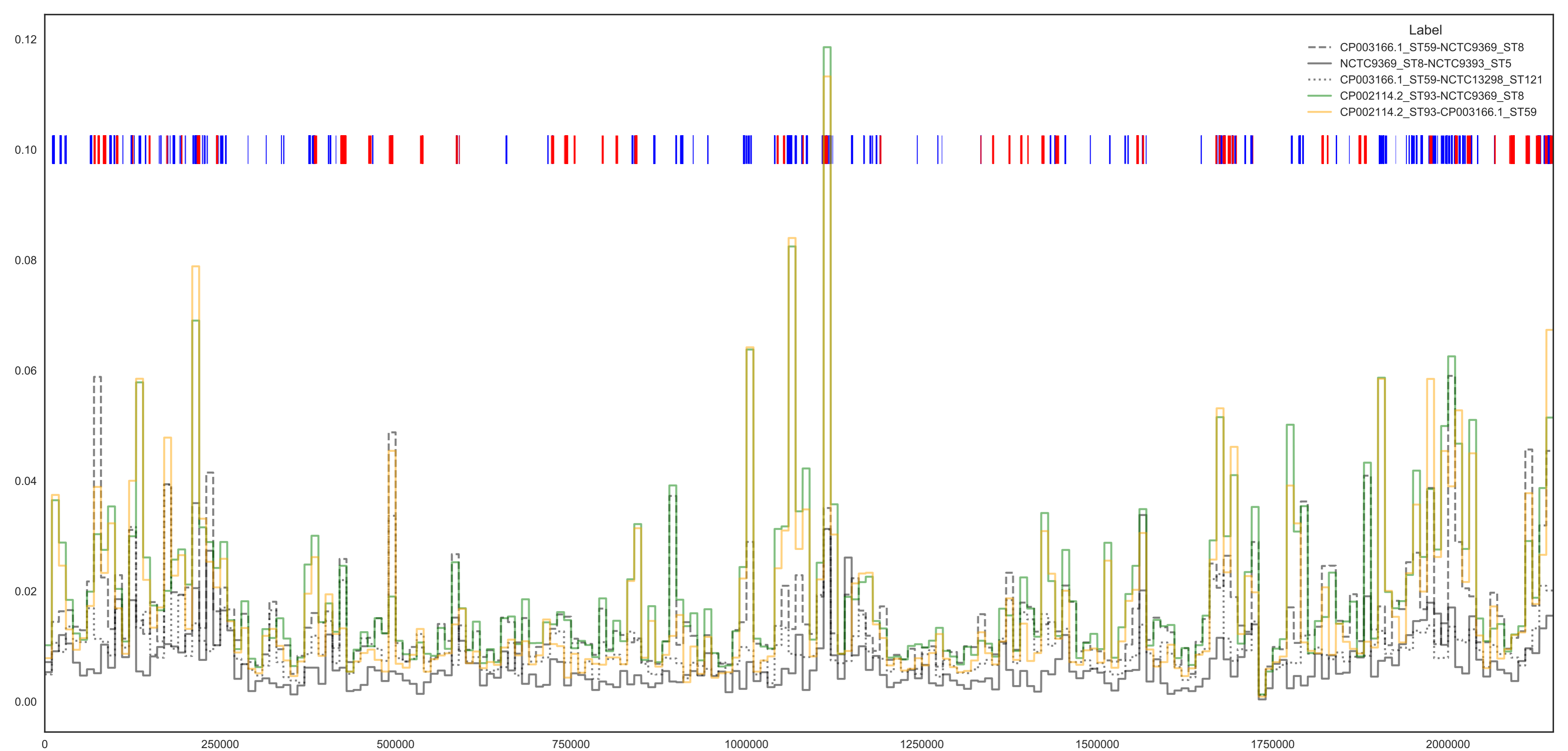

Supplement: FIGURE S3 — Additional pair-wise nucleotide divergence comparisons between representative S. aureus genomes including ST93. Pair-wise nucleotide divergence across a 10,000 bp sliding window along the S. aureus core genome with comparisons between ST59-ST121, ST93-ST59, ST93-ST8, ST59-ST8, and ST8-ST5. Bases affected by recombination as detected by Gubbins across the ST93 genome are represented above with shared ancestral events (red) and events unique to the ST93 terminal branch (blue). Terminal branch recombination regions align with genomic positions where ST93 is highly diverged compared to other S. aureus. [file Image_3.PDF]

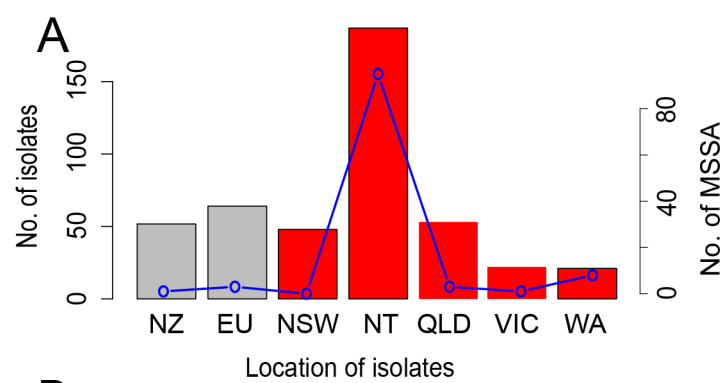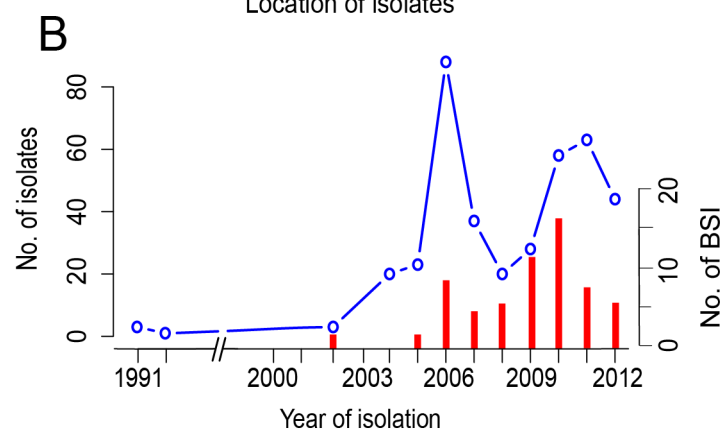

Supplement: FIGURE S4 — Source of isolates. (A) Depicts the location from where the isolates originated with red boxes representing states and territories of Australia. European countries include England (n = 45), Scotland (n = 12), Denmark (n = 4), France (n = 1), and Italy (n = 1). Note, Samoan isolates (n = 4) are not depicted. The number of MSSA isolates sequenced, by location are shown on the secondary axis by the blue line. (B) Represents the temporal distribution of the isolates with the number of blood stream isolates depicted by the red bars on the secondary axis. Note that data on source of infection was unknown for 52 isolates. [file Image_4.PDF]

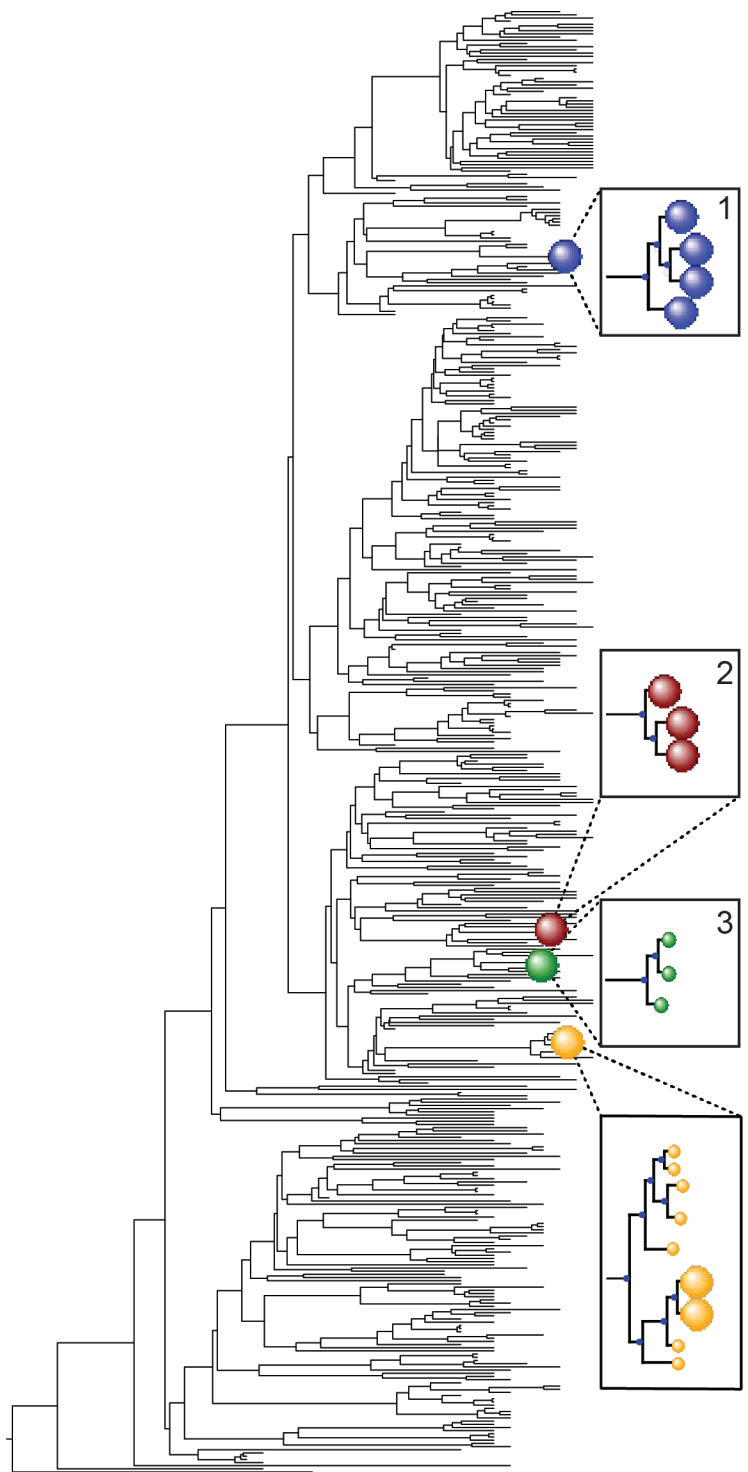

Supplement: FIGURE S5 — Evidence of short transmission chains in the United Kingdom. Maximum clade credibility tree as in Figure 1, but with transmission chains from the United Kingdom highlighted. Box 1 – four isolates from three individuals of which two were siblings. Box 2 – three isolates from separate patients in same pediatric hospital. Box 3 – there were no obvious epidemiological links with cases geographically widely distributed. Box 4 – there were no obvious epidemiological links. [file Image_5.PDF]

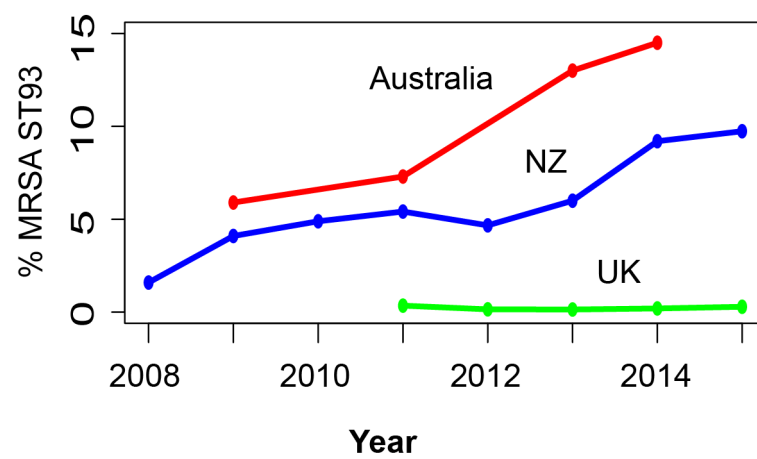

Supplement: FIGURE S6 — Proportion of methicillin-resistant Staphylococcus aureus that are ST93-MRSA. This represents longitudinal data of ongoing MRSA typing data from Australia, New Zealand (NZ) and United Kingdom (UK). Graph depicts the percentage of MRSA isolates that are ST93 between 2008 and 2015. Data were obtained from Public Health England, United Kingdom, the Institute of Environmental Science and Research, New Zealand, and the Australian Collaborating Centre for Enterococcus and Staphylococcus Species (ACCESS) Typing and Research, Australia. [file Image_6.PDF]

**B**

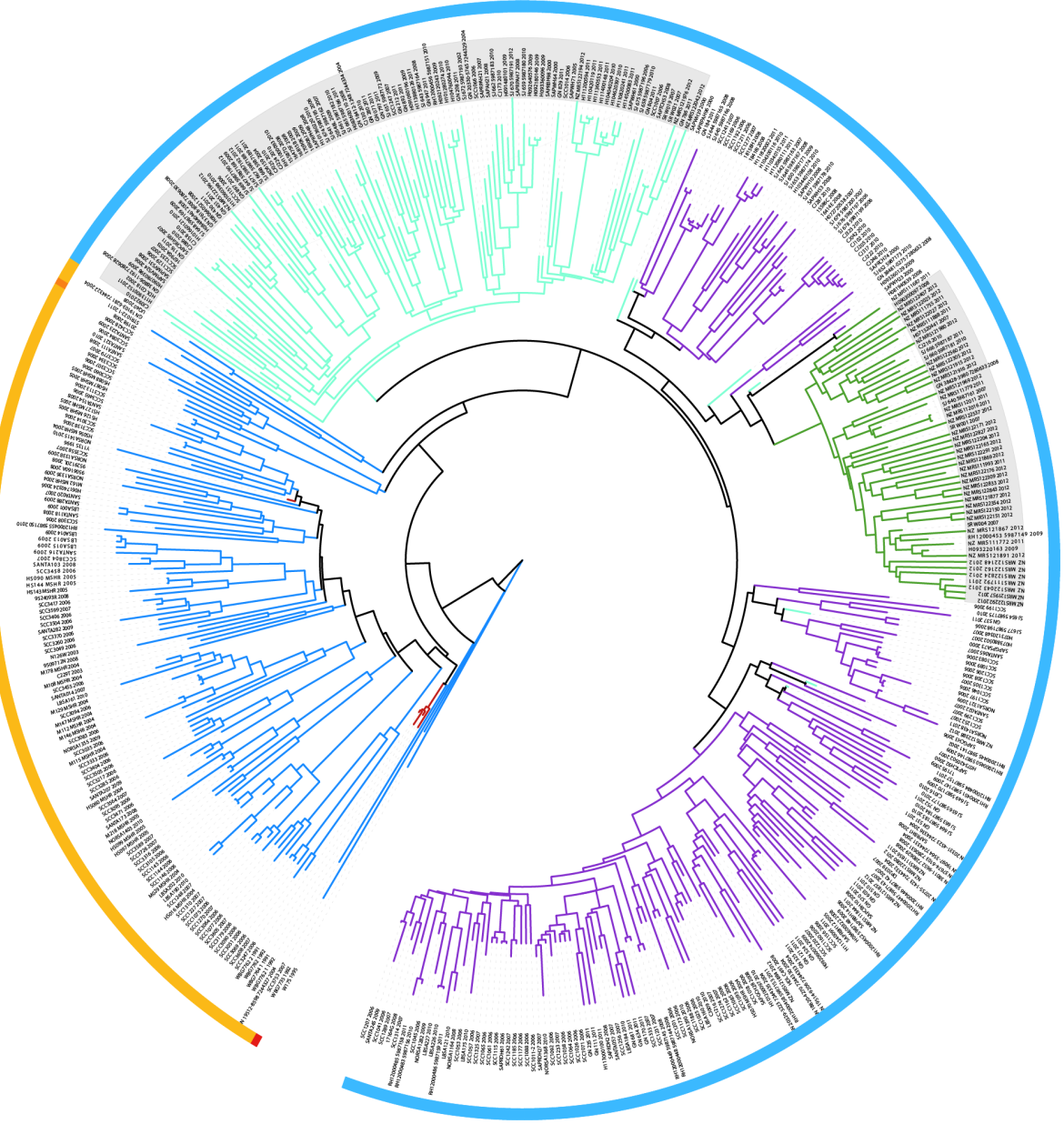

**A**

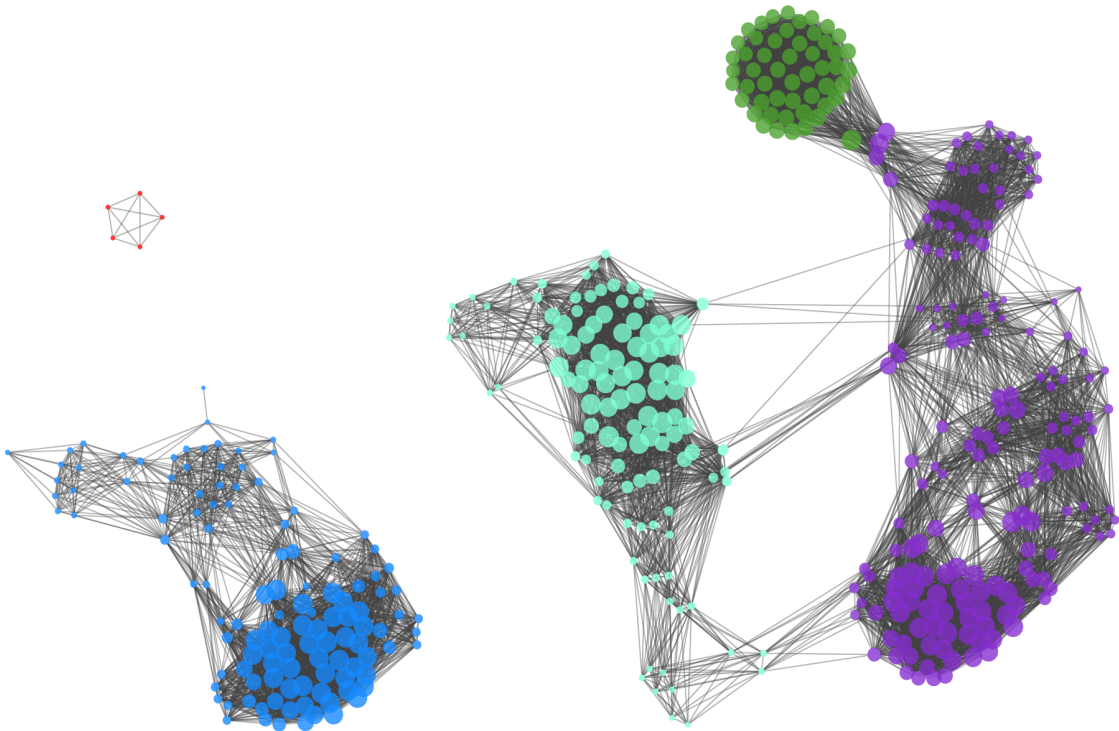

Supplement: FIGURE S7 — Network clusters of ST93. (A) General network clusters using the low-resolution fast-greedy modularity optimization algorithm. (B) Cluster assignments were mapped back to the phylogeny. [file Image_7.pdf]
